# A muscular hypotonia-associated STIM1 mutant at R429 induces abnormalities in intracellular Ca^2+^ movement and extracellular Ca^2+^ entry in skeletal muscle

**DOI:** 10.1038/s41598-019-55745-z

**Published:** 2019-12-16

**Authors:** Jun Hee Choi, Mei Huang, Changdo Hyun, Mi Ri Oh, Keon Jin Lee, Chung-Hyun Cho, Eun Hui Lee

**Affiliations:** 1 Department of Physiology, College of Medicine, The Catholic University of Korea, Seoul, 06591 Korea; 2 Department of Biomedicine & Health Sciences, Graduate School, The Catholic University of Korea, Seoul, 06591 Korea; 3 Department of Biomedical Sciences and Pharmacology, College of Medicine, Seoul National University, Seoul, 03080 Republic of Korea

**Keywords:** Mechanisms of disease, Calcium and vitamin D, Calcium signalling, Calcium channels

## Abstract

Stromal interaction molecule 1 (STIM1) mediates extracellular Ca^2+^ entry into the cytosol through a store-operated Ca^2+^ entry (SOCE) mechanism, which is involved in the physiological functions of various tissues, including skeletal muscle. STIM1 is also associated with skeletal muscle diseases, but its pathological mechanisms have not been well addressed. The present study focused on examining the pathological mechanism(s) of a mutant STIM1 (R429C) that causes human muscular hypotonia. R429C was expressed in mouse primary skeletal myotubes, and the properties of the skeletal myotubes were examined using single-cell Ca^2+^ imaging of myotubes and transmission electron microscopy (TEM) along with biochemical approaches. R429C did not interfere with the terminal differentiation of myoblasts to myotubes. Unlike wild-type STIM1, there was no further increase of SOCE by R429C. R429C bound to endogenous STIM1 and slowed down the initial rate of SOCE that were mediated by endogenous STIM1. Moreover, R429C increased intracellular Ca^2+^ movement in response to membrane depolarization by eliminating the attenuation on dihydropyridine receptor-ryanodine receptor (DHPR-RyR1) coupling by endogenous STIM1. The cytosolic Ca^2+^ level was also increased due to the reduction in SR Ca^2+^ level. In addition, R429C-expressing myotubes showed abnormalities in mitochondrial shape, a significant decrease in ATP levels, and the higher expression levels of mitochondrial fission-mediating proteins. Therefore, serial defects in SOCE, intracellular Ca^2+^ movement, and cytosolic Ca^2+^ level along with mitochondrial abnormalities in shape and ATP level could be a pathological mechanism of R429C for human skeletal muscular hypotonia. This study also suggests a novel clue that STIM1 in skeletal muscle could be related to mitochondria via regulating intra and extracellular Ca^2+^ movements.

## Introduction

Excitation-contraction (EC) coupling is a prerequisite for skeletal muscle contraction^1–3^. During skeletal EC coupling, action potential in mature skeletal muscle cells by neural stimulation reaches transverse (t)-tubule. The action potential activates dihydropyridine receptors (DHPRs, voltage-gated Ca^2+^ channels (Ca_v_1.1.)) in the t-tubule membrane. Ryanodine receptor 1 molecules (RyR1s, internal Ca^2+^ channels) on the membrane of the sarcoplasmic reticulum (SR, corresponding to ER in other cell types) are subsequently opened due to the physical interactions with the active DHPRs (i.e., DHPR-RyR1 coupling), which represents a key step in inducing intracellular Ca^2+^ movement that is required for skeletal muscle contraction. These interactions allow Ca^2+^ movement from the SR to the cytosol through RyR1s (i.e., intracellular Ca^2+^ movement). The Ca^2+^ ions from the SR are major sources of Ca^2+^ for skeletal muscle contraction and serve as critical switches that turn on cross-bridge cycles of skeletal muscle. Relaxation of skeletal muscle involves Ca^2+^-reuptake from the cytosol to the SR^2–4^. Sarcoplasmic/endoplasmic reticulum Ca^2+^-ATPase (SERCA) is responsible for Ca^2+^-reuptake and SERCA1a is the major adult isoform^1–4^. The efficient arrangement of DHPR, RyR1, SERCA1a, and their regulatory proteins in a triad junction is crucial for their proper functions and intermolecular interactions^2,3^. Junctophilin (JP) and mitsugumin 29 (MG29) participate in the development of the triad junction during the terminal differentiation of myoblasts (proliferative forms of satellite cells) to myotubes (mature forms of skeletal muscle cells) in the tissue^2,3,5,6^. Various myogenic factors exert their influence to different stages of terminal differentiation, such as a higher expression level of myogenin or myosin heavy chain (MHC) and a lower expression level of MyoD at the late stage of terminal differentiation^7,8^. Overall, spatiotemporal distributions of intracellular Ca^2+^ ions, timely expression of myogenic factors, proper positioning of the proteins mentioned above in the triad junction of skeletal muscle are important factors for contraction and relaxation. A failure at any point induces defective skeletal muscle function such as life-threatening malignant hyperthermia^9^.

Store-operated Ca^2+^ entry (SOCE) is a Ca^2+^ entryway for the sustained presence of Ca^2+^ in the cytosol to achieve strong and durable cytosolic Ca^2+^ signals in most cell types^10–12^. The main SOCE-mediating proteins are stromal interaction molecule 1 (STIM1, a Ca^2+^ sensor) in the ER membrane and Orai1 (an extracellular Ca^2+^ entry channel) in the plasma membrane^10–12^. During SOCE, STIM1 senses the waste of Ca^2+^ in the ER (i.e., ER depletion). This event allows the movement of STIM1s to the ER membranes that are close to the plasma membranes where STIM1s interact with Orai1s to form puncta^13–17^. During the formation of puncta, STIM1 self-oligomerizes via its EF–sterile α-motif (EF-SAM) domain, first coiled-coil (CC) domain, and/or Ca^2+^ release-activated Ca^2+^-activating domain (CAD)^15,16^. Punctum formation activates the entry of extracellular Ca^2+^ through Orai1 into the cytosol. The cytosolic Ca^2+^ level that is elevated by SOCE returns to a normal level via the import of cytosolic Ca^2+^ to the SR by SERCAs or the export of cytosolic Ca^2+^ to the extracellular area by plasma membrane Ca^2+^ ATPases (so called PMCAs)^18^. In skeletal muscle, interestingly, puncta appear during the terminal differentiation in an SR depletion-independent manner^12,19,20^. This is the reason for a more rapid SOCE in skeletal muscle than in other cells types. In addition to the main role of Orai1 in SOCE^2,21^, canonical-type transient receptor potential cation channels (TRPCs) participate in SOCE-mediated Ca^2+^ entry in skeletal muscle^22–24^. Skeletal muscle expresses TRPC1, TRPC3, TRPC4, and TRPC6^25^. Interestingly, STIM1 plays other important roles in skeletal muscle. Via a direct interaction to DHPR, STIM1 attenuates DHPR-RyR1 coupling that is required for skeletal muscle contraction^20^. Similarly, suppressive activity of STIM1 on another type of voltage-gated Ca^2+^ channel (Ca_v_1.2) has been reported^26^. STIM1 is also participates in maintaining the full activity of SERCA1a for the relaxation of skeletal muscle^27^.

The conditional deletion of STIM1 in mouse skeletal muscle results in no mediation of SOCE, rapid fatigue, and perinatal death from skeletal myopathy^28^. Muscle fibres from a *mdx* mouse (an animal model of Duchenne muscular dystrophy) show increases in SOCE as well as STIM1 expression^29,30^. Patients with mutations in STIM1 show the following pathological skeletal muscle conditions: congenital and global muscular hypotonia showing a decrease in muscle tone and progressive muscular dystrophy by a loss-of-function mutation (E136X)^20,31,32^, muscular atrophy, tubular aggregate myopathy, and/or progressive muscle weakness by STIM1 missense mutations (H72Q, D84G, H109N or H109R)^20,33^. A point mutation at R429 of STIM1 (R429C) has been reported in human patients with insufficient immunity and muscular hypotonia^34^. The abolishment of SOCE by the presence of R429C in T cells is thought to cause insufficient immunity in patients^34,35^. However, the pathological mechanism(s) of muscular hypotonia in patients with R429C have not yet been well addressed. Considering that various mutations in STIM1 cause the human skeletal muscle diseases mentioned above, examining the pathological effect(s) of R429C on the major functions of skeletal muscle, such as intracellular Ca^2+^ movement, which is needed for skeletal muscle contraction, is important and helpful in understanding the multiple physiological roles of STIM1 in skeletal muscle.

## Results

### R429C also does not mediate SOCE in skeletal myotubes

**Figure 1:**
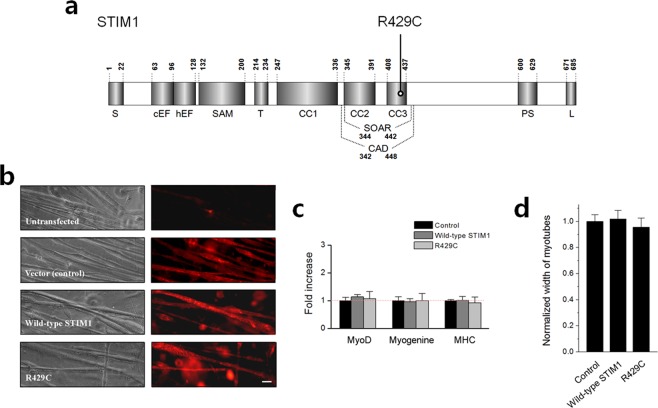
Schematic of the primary structure of STIM1 and the expression of R429C in mouse primary skeletal myotubes. (**a**) Each domain of STIM1 is presented according to previous reports on the overall structure^66^, CAD/SOAR^13,14,67^, and CC domains^35^. The location of R429C is indicated. Numbers indicate the amino acid sequence. S, signal peptide; cEF, canonical EF-hand; hEF, non-functional hidden EF-hand; SAM, sterile α-motif; T, transmembrane domain; CC, coiled-coil domain; CAD/SOAR, Ca^2+^ release-activated Ca^2+^-activating domain/STIM1-Orai1-activating region; PS, proline/serine-rich domain; and L, lysine-rich domain. (**b**) Mouse primary skeletal myotubes that were untransfected or transfected with either cDNA of empty vector, wild-type STIM1, or R429C were stained with anti-GFP (for detecting CFP or CFP-tagged proteins) and Cy3-conjugated secondary antibodies. The bar represents 100 µm. (**c**) mRNA levels of MyoD, myogenin, and MHC in myotubes were analyzed by qRT-PCR. ‘Control’ means myotubes that were transfected with cDNA of empty vector (also for subsequent experiments). The normalized mean values of each to the mean value of the control are summarized as histograms. Difference was considered to be considerable at more than 2-fold increase and there was no considerable difference. The values are presented as the mean ± s.d. for triple experiments (Supplementary Table S1). (**d**) The width of myotubes was measured. The normalized mean values of each to the mean value of the control are summarized as histograms. Significant difference compared with the control (*p* < 0.05) and there was no significant difference in the width of the myotubes. The values are presented as the mean ± s.e.m. for the number of myotubes shown in parentheses in Table 1.

To study the pathological role(s) of R429C in skeletal muscle (Fig. 1a), R429C was expressed in mouse primary skeletal myotubes rather than in heterologous expression systems in order to avoid possible artefacts introduced by the cell system (Fig. 1b). To evaluate the degree of terminal differentiation of myoblasts to myotubes, mRNA levels of myogenic factors such as MyoD, myogenin, and MHC in the myotubes were examined using quantitative real-time PCR (qRT-PCR) (Fig. 1c). Myotubes that were transfected with empty vector were used as a control (also for subsequent experiments). There was no considerable difference in their mRNA levels by the expression of R429C. In addition, the width of myotubes (i.e., representing the degree of terminal differentiation) was measured (Fig. 1d). No significant difference was induced in the widths of myotubes by the expression of R429C. Therefore R429C-expressing myotubes did not show a significant difference in myotube formation compared with the vector control or wild-type STIM1. This suggests that STIM1 is not a critical protein for the terminal differentiation of skeletal muscle.

**Figure 2:**
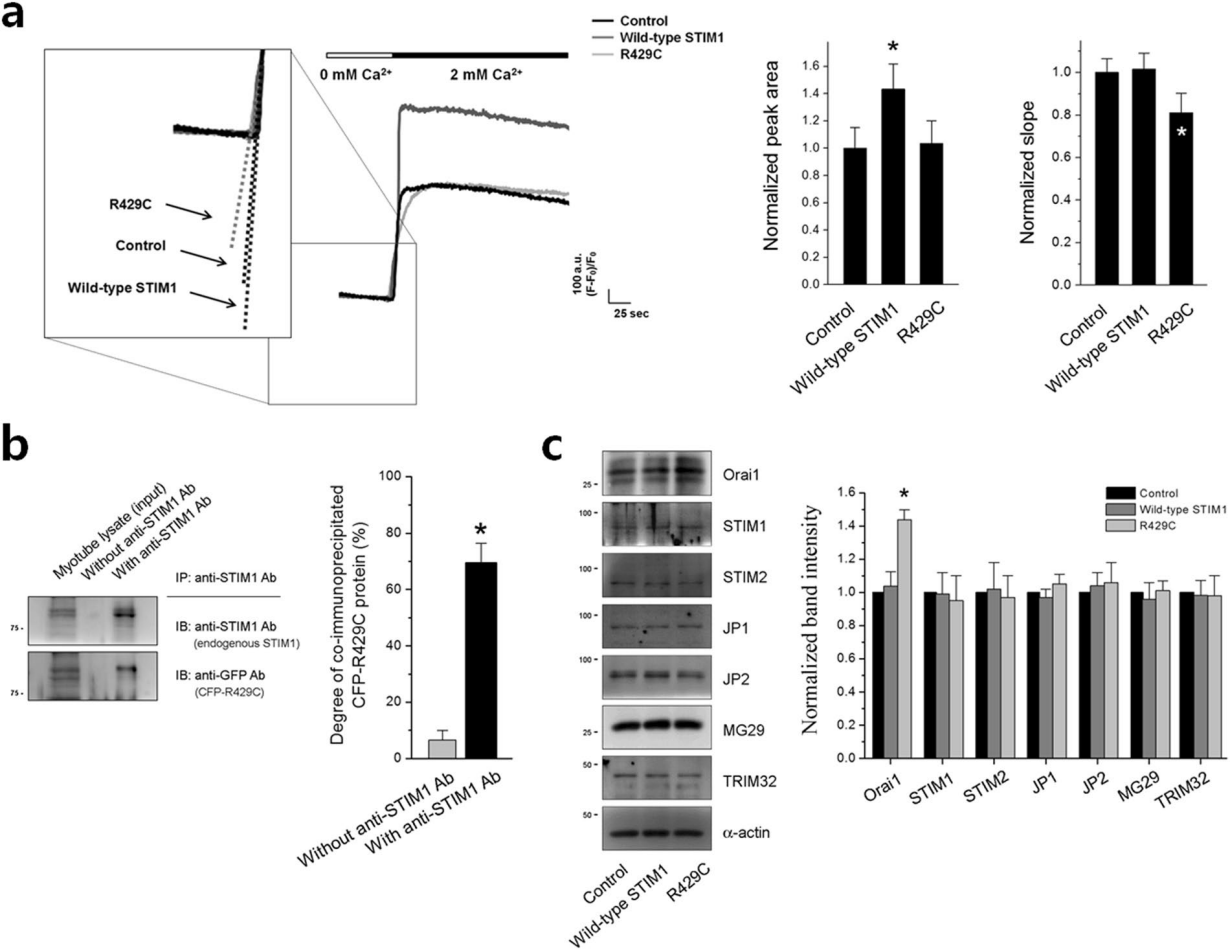
SOCE, co-immunoprecipitation of endogenous STIM1 with R429C, and expression levels of various proteins. (**a**) The Ca^2+^ in the SR of R429C-expressing myotubes was depleted by treatment with TG (2.5 μM) in the absence of extracellular Ca^2+^, and extracellular Ca^2+^ (2 mM) was then applied to the myotubes to induce SOCE. A representative trace for each group is shown. The boxed area was enlarged and dotted lines indicate the slopes in the rising phase of SOCE. The results are summarized as histograms for the area under the peaks (left-hand side) or for the slope (right-hand side). The experimental mean values were normalized to the control mean values. The values are presented as the mean ± s.e.m. for the number of myotubes shown in the parentheses in Table 1. (**b**) Co-immunoprecipitation assay of endogenous STIM1 with R429C was conducted using the lysate of R429C-expressing myotubes and anti-STIM1 antibody. Myotube lysate refers to the lysate of R429C-expressing myotubes. A representative result is presented. In the lane of myotube lysate, upper bands are CFP-R429Cs and lower bands are endogenous STIM1s. Degree of co-immunoprecipitated R429C to the corresponding total protein is presented as histograms in the right-hand side. *Significant difference was compared with ‘without anti-STIM1 Ab’ (*p* < 0.05). (**c**) The lysate of R429C-expressing myotubes was subjected to immunoblot assays with antibodies against the seven indicated proteins. *α*-actin was used as a loading control. A representative result is presented. The expression level of each protein normalized to the mean value of the control is presented as histograms on the right-hand side. The values in (**b**,**c**) are presented as the mean ± s.e.m. for the number of experiments shown in the parentheses of Supplementary Tables S3 and S4. *Significant difference compared with the control (*p* < 0.05). The blots that were cropped from different gels were grouped in (**b**,**c**), and the full-length blots are presented in Supplementary Figs. S4 and S5.

To examine the effect of R429C on SOCE in skeletal muscle, Ca^2+^ in the SR of R429C-expressing myotubes was depleted with thapsigargin (TG) in the absence of extracellular Ca^2+^ to avoid extracellular Ca^2+^ entry during depletion, and extracellular Ca^2+^ was then applied to the myotubes to measure SOCE. Wild-type STIM1 enhanced SOCE. However, SOCE was not enhanced by R429C, suggesting that R429C does not mediate SOCE in skeletal muscle (Fig. 2a, left histograms and Table 1), as was previously found in a heterologous expression system (HEK293 cells)^35^.

**Table 1:** Properties of skeletal myotubes expressing wild-type STIM1 or R429C.

		Control	Wild-type STIM1	R429C
Width of myotubes		1.00 ± 0.05 (40)	1.02 ± 0.07 (40)	0.95 ± 0.07 (40)
SOCE	Peak area	1.00 ± 0.15 (36)	1.43 ± 0.18 * (36)	1.04 ± 0.16 (39)
	Slope (initial rate)	1.00 ± 0.06 (30)	1.02 ± 0.08 (30)	0.81 ± 0.09 * (30)
KCl response		1.00 ± 0.07 (54)	0.79 ± 0.08 * (54)	1.20 ± 0.10 * (72)
Caffeine response		1.00 ± 0.07 (81)	1.01 ± 0.11 (57)	0.99 ± 0.05 (57)
Cytosolic [Ca^2+^] (nM)		84.37 ± 4.67 (69)	85.84 ± 5.99 (70)	101.19 ± 8.07 * (70)
Ca^2+^ level in the SR		1.00 ± 0.09 (50)	1.00 ± 0.10 (50)	0.80 ± 0.10 * (50)
Total cellular Ca^2+^ level		1.00 ± 0.09 (36)	0.98 ± 0.09 (34)	1.03 ± 0.11 (38)

The values, except for those of cytosolic [Ca^2+^], were normalized to the mean values of the controls. The values are presented as the mean ± s.e.m. for the number of myotubes shown in parentheses. *Significant difference compared with the control (*p* < 0.05).

Interestingly, R429C-expressing myotubes showed another property. The slope at the rising phase of SOCE in R429C-expressing myotubes was significantly less steep (i.e., slower rising) than those in control or wild-type STIM1 (Fig. 2a, right histograms and Table 1). Considering that R429C does not mediate SOCE, the less steep slope of SOCE means that SOCE by endogenous STIM1 (i.e., endogenous SOCE) was also affected by R429C. According to the previous reports that STIM1 forms oligomers during SOCE, the binding ability of R429C to endogenous STIM1 was examined by co-immunoprecipitation assay using the lysate from R429C-expressing myotubes (Fig. 2b). R429C was significantly co-immunoprecipitated with endogenous STIM1. This result supports that R429C could slow down the initial rate of endogenous SOCE by binding to endogenous STIM1, which is a dominant-negative effect of the R429C mutant over endogenous STIM1. CFP tag itself did not bind to endogenous STIM1 (Supplementary Fig. S1). Therefore these results suggest that R429 of STIM1 is a critical residue for the mediation of SOCE in skeletal muscle.

To assess the expression level of seven proteins that mediate or regulate SOCE in skeletal muscle, immunoblot assays were conducted with the lysate of R429C-expressing myotubes (Fig. 2c). There was no significant change in the expression level of endogenous STIM1 or STIM2. Interestingly the expression of endogenous Orai1 was increased by R429C, despite no mediation of SOCE by R429C. Therefore, no mediation of SOCE by R429C was not due to a simple decrease in the expression level of endogenous STIM1, STIM2, or Orai1. There was no significant change in the expression level of tripartite motif-containing protein 32 (TRIM32) that controls myogenic differentiation via regulating c-Myc and is related to human limb-girdle muscular dystrophy 2 H^36–38^. In addition, there was also no significant change in the expression level of TRPC1, TRPC3, TRPC4, or TRPC6, which are known to mediate extracellular Ca^2+^ entry in skeletal muscle (Supplementary Fig. S2). The expression levels of other Ca^2+^-related proteins in skeletal muscle, such as JP1, JP2, MG29, calsequestrin 1, MG53, or calmodulin 1, were also not significantly changed (Fig. 2c and Supplementary Fig. S2). These results suggest that neither the increase in intracellular Ca^2+^ movement nor no enhancement of SOCE in R429C-expressing myotubes was caused by a change in the expression level of proteins that mediate or regulate skeletal muscle function but were instead due to the mutation at R429.

### R429C increases intracellular Ca^2+^ movement by eliminating the attenuation on DHPR-RyR1 coupling by endogenous STIM1 in skeletal myotubes

**Figure 3:**
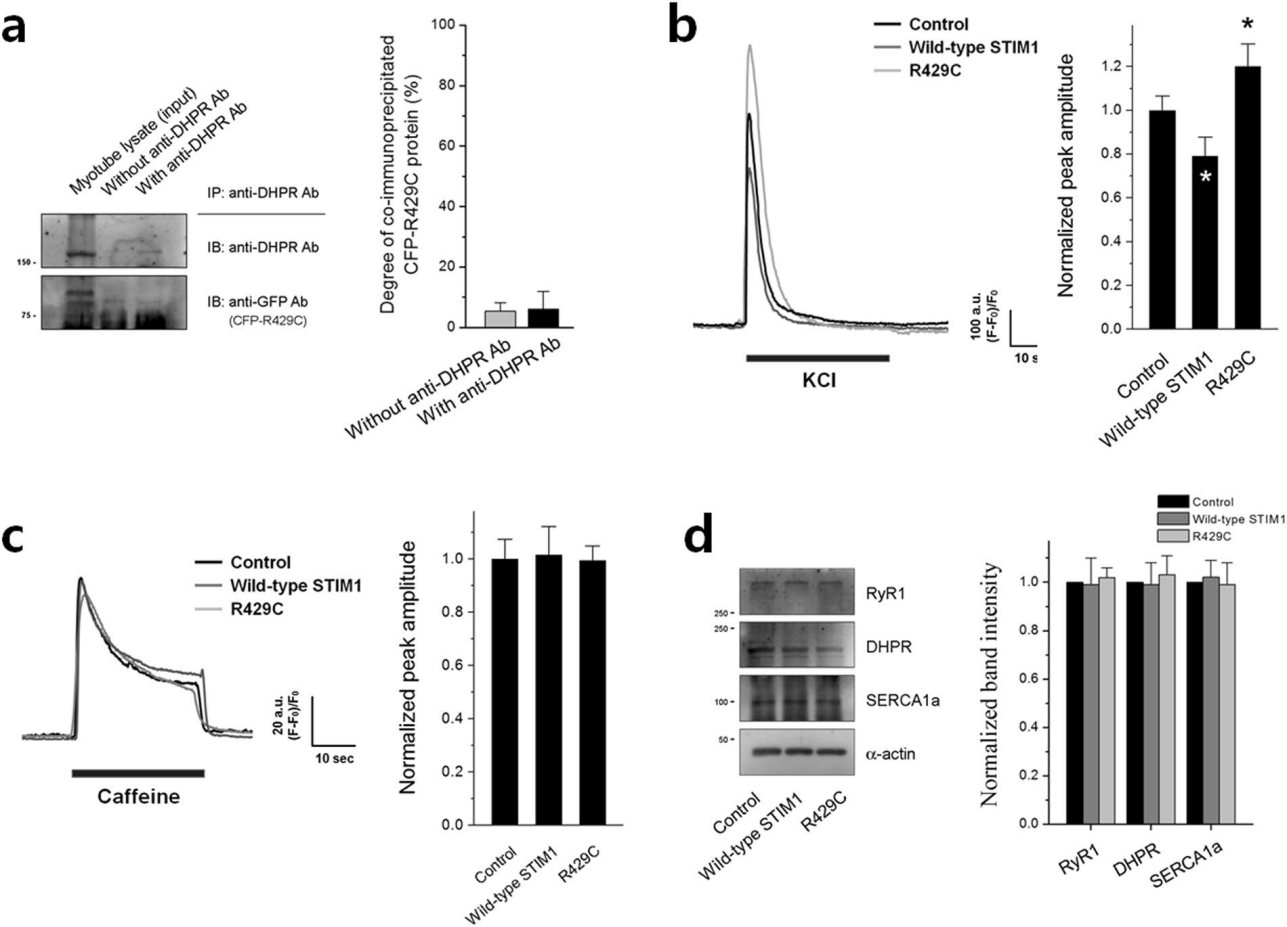
Intracellular Ca^2+^ movements. (**a**) Co-immunoprecipitation assay of DHPR with R429C was conducted using the lysate of R429C-expressing myotubes and anti-DHPR antibody. Myotube lysate refers to the lysate of R429C-expressing myotubes. A representative result is presented. Degree of co-immunoprecipitated R429C to the corresponding total protein is presented as histograms in the right-hand side. Significant difference was compared with ‘Without anti-DHPR Ab’ (*p* < 0.05) and there was no significant difference. The values are presented as the mean ± s.e.m. for the number of experiments shown in the parentheses of Supplementary Table S4. KCl (**b**) or caffeine (**c**) was applied to R429C-expressing myotubes, and intracellular Ca^2+^ movement from the SR to cytosol through RyR1 was measured. A representative trace for each group is shown. Histograms on the right-hand side show the peak amplitude normalized to the mean value of the control. The results are presented as the mean ± s.e.m. for the number of experiments shown in parentheses in Table 1. *Significant difference when compared with the control (*p* < 0.05). (**d**) The lysate of R429C-expressing myotubes was subjected to immunoblot assays with antibodies against RyR1, DHPR, or SERCA1a. *α*-actin was used as a loading control. The expression level of each protein normalized to the mean value of the control is presented as histograms on the right-hand side. Significant difference compared with the control (*p* < 0.05) and there was no significant difference. The values in (**a**) and (**d**) are presented as the mean ± s.e.m. for the number of experiments shown in the parentheses of Supplementary Tables S3 and S4, respectively. The blots that were cropped from different gels were grouped in (**a**) and (**d**), and the full-length blots are presented in Supplementary Figs. S6 and S7.

Based on our previous report that STIM1 attenuates DHPR-RyR1 coupling required for skeletal muscle contraction by binding to DHPR^20^, the binding ability of R429C to DHPR was examined by co-immunoprecipitation assay using lysate from R429C-expressing myotubes (Fig. 3a and Supplementary Fig. S3). R429C did not bind to DHPR. Considering the interaction between R429C and endogenous STIM1 in Fig. 2b, it is possible that the attenuation of DHPR-RyR1 coupling by endogenous STIM1 is eliminated by the interaction between R429C and endogenous STIM1.

To test this possibility, a membrane depolarizer (KCl) that induces membrane depolarization such as the one during EC coupling was applied to R429C-expressing myotubes, and intracellular Ca^2+^ movements from the SR to the cytosol through RyR1 were measured using single-cell Ca^2+^ imaging of myotubes. Intracellular Ca^2+^ movement in response to membrane depolarization in skeletal myotubes mimics intracellular Ca^2+^ movement required for skeletal muscle contraction. As shown in our previous report^20^, wild-type STIM1 induced a significant decrease in Ca^2+^ movement in response to membrane depolarization compared with that in the control due to the attenuation of DHPR-RyR1 coupling by wild-type STIM1 (Fig. 3b and Table 1). However, R429C increased Ca^2+^ movement in response to membrane depolarization. This supports the theory that the attenuation of DHPR-RyR1 coupling by endogenous STIM1 is eliminated by R429C, which is a dominant-negative effect of the R429C mutant over endogenous STIM1.

It is possible that the increase in intracellular Ca^2+^ movement by R429C was simply due to a change in RyR1 activity rather than a change in DHPR-RyR1 coupling. To examine this scenario, RyR1 activity was assessed by applying a direct agonist of RyR1 (caffeine^39^) to R429C-expressing myotubes (Fig. 3c and Table 1). Ca^2+^ movement in response to caffeine was not significantly changed by R429C, suggesting that the overall activity of RyR1 was not changed by R429C. Therefore, the possible change in RyR1 activity was ruled out. In addition, the possibility that an increase or decrease in the expression level of DHPR, RyR1, or SERCA1a as the cause of the increase in Ca^2+^ movement by R429C was also ruled out (Fig. 3d, no significant changes in the expression levels).

### R429C elevates the cytosolic Ca^2+^ level and induces mitochondrial abnormalities in skeletal myotubes

**Figure 4:**
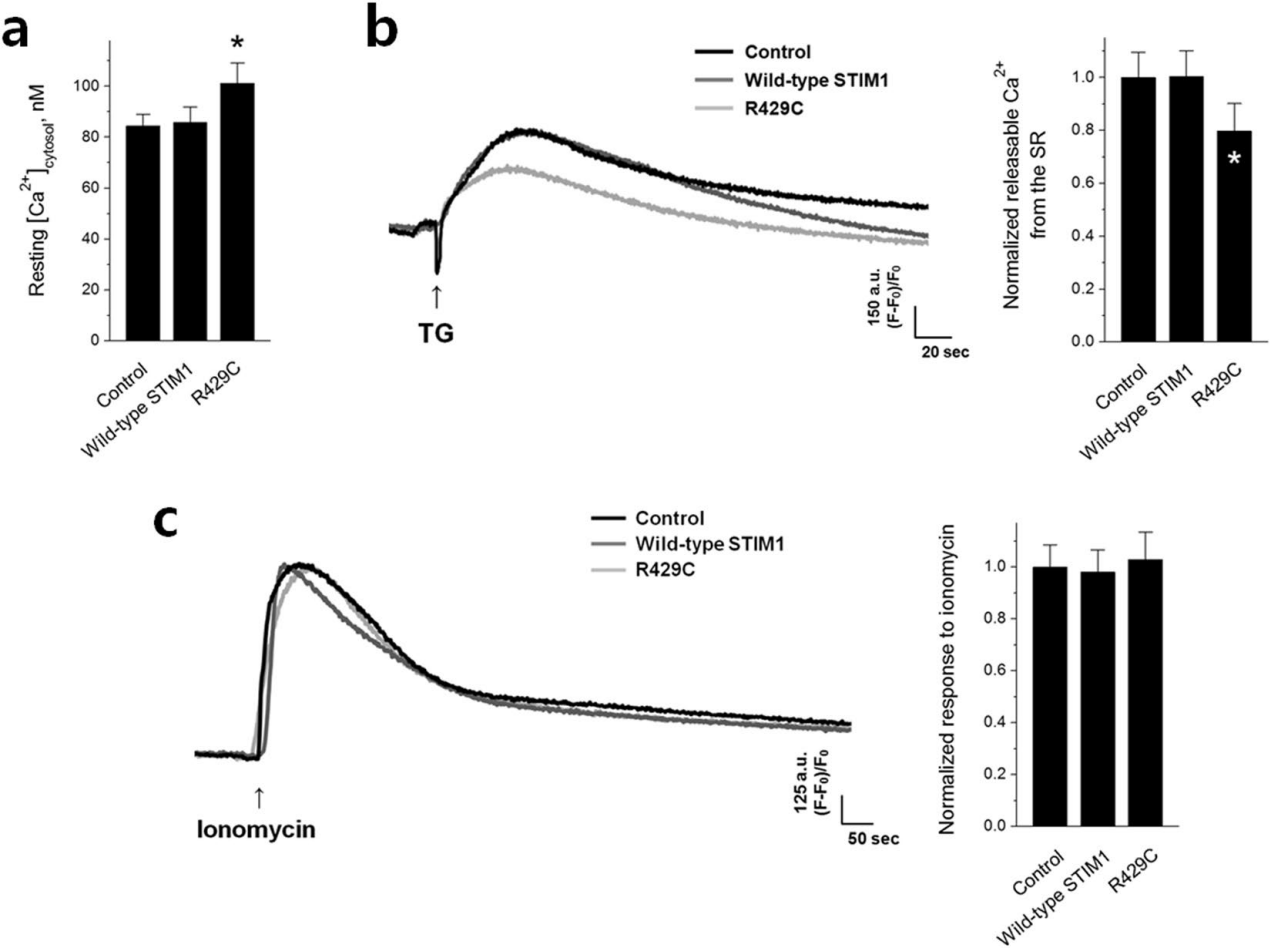
Cytosolic Ca^2+^ levels, releasable Ca^2+^ from the SR, and total cellular Ca^2+^ levels. (**a**) Cytosolic Ca^2+^ levels at rest were measured in R429C-expressing myotubes, and the mean values are summarized as histograms. (**b**) Releasable Ca^2+^ from the SR to the cytosol was measured by treatment with TG (2.5 μM) in the absence of extracellular Ca^2+^. (**c**) Total cellular Ca^2+^ levels were measured by treatment with ionomycin (5 μM) in the absence of extracellular Ca^2+^. A representative trace for each group is shown in. (**b**,**c**) The normalized mean values of each to the mean value of the control are summarized as histograms on the right-hand side. The values are presented as the mean ± s.e.m. for the number of myotubes shown in parentheses in Table 1. *Significant difference compared with the control (*p* < 0.05).

To examine the aftereffects of no mediation of SOCE and the increase in intracellular Ca^2+^ movement by R429C, first, the cytosolic Ca^2+^ level at rest was measured in R429C-expressing myotubes (Fig. 4a and Table 1). Cytosolic Ca^2+^ levels were significantly increased by R429C. To address the reason for this increase, the releasable Ca^2+^ from the SR (which allows estimation of the Ca^2+^ level in the SR) was measured by depleting the SR with TG in the absence of extracellular Ca^2+^ (Fig. 4b and Table 1). The absence of extracellular Ca^2+^ allows an assessment of releasable Ca^2+^ exclusively from the SR without extracellular Ca^2+^ entry during SR depletion. Interestingly, the Ca^2+^ level in the SR was significantly decreased by R429C, without a significant change in the total cellular Ca^2+^ level (Fig. 4c and Table 1). Therefore, the increase in the cytosolic Ca^2+^ level by R429C could be mainly due to the reduction in Ca^2+^ in the SR.

**Figure 5:**
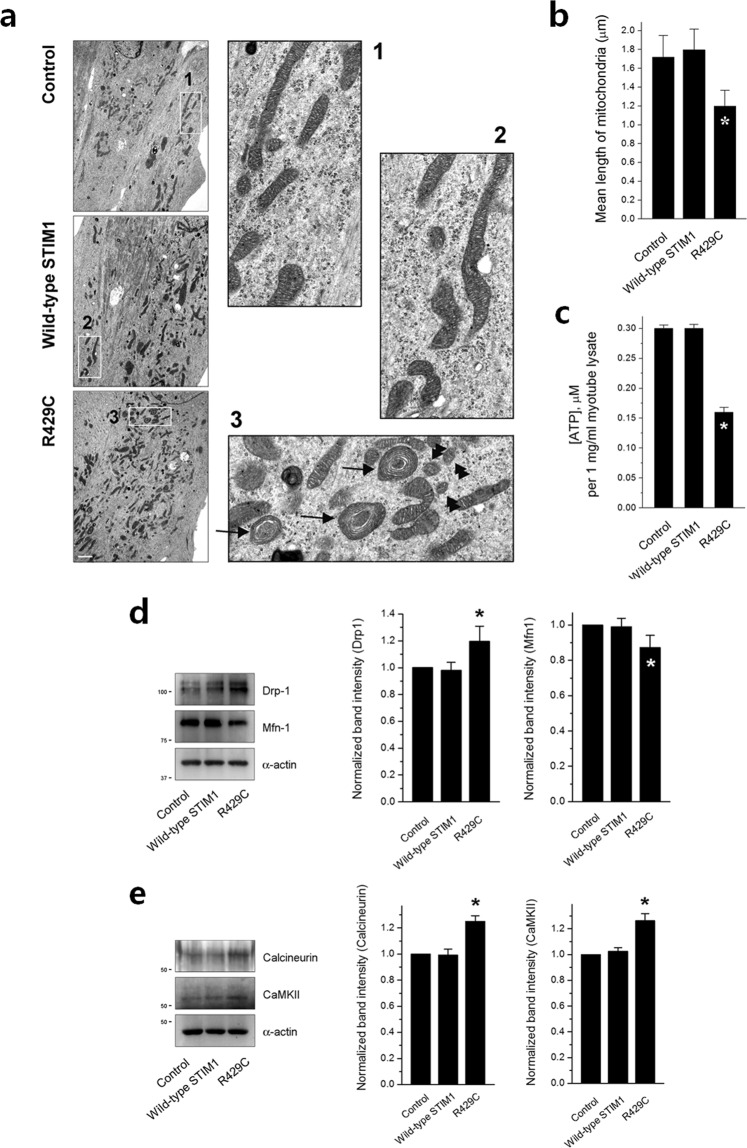
Mitochondrial shape, ATP level, and expression level of mitochondrial fusion- or fission-related proteins. (**a**) Mitochondria in R429C-expressing myotubes were observed using TEM. Areas in the numbered boxes (from 1 to 3) were enlarged. Mitochondria with onion-shaped cristae in R429C-expressing myotubes are indicated by black arrows in the enlarged image 3. Representative small mitochondria in R429C-expressing myotubes are indicated by black double arrowheads in the enlarged image 3. The bar represents 2 µm. (**b**) The length of mitochondria with normal shapes (except mitochondria with onion-shaped cristae) was measured in the unit area (37.5 × 25 µm^2^). The results are presented as histograms. The values are presented as the mean ± s.e.m. for the unit areas shown in parentheses in Table 2. (**c**) ATP levels in R429C-expressing myotubes were measured using the lysate of R429C-expressing myotubes (1 mg/ml). The lysate of R429C-expressing myotubes was subjected to immunoblot assays with antibodies against mitochondrial fusion- or fission-mediating proteins (**d**) or regulatory proteins (**e**). *α*-actin was used as a loading control. The normalized expression level of each to the mean value of the control is presented in histograms on the right-hand side. The values in (**c**), (**d**,**e**) are presented as the mean ± s.e.m. for three independent experiments (Table 2). *Significant difference compared with the control (*p* < 0.05). The blots that were cropped from different gels were grouped in (**d**,**e**), and the full-length blots are presented in Supplementary Figs. S8 and S9.

To assess the effect of the increases in intracellular Ca^2+^ movement and cytosolic Ca^2+^ levels by R429C on other aspects, R429C-expressing myotubes were observed by TEM. Dramatic abnormalities in the shape of mitochondria were found in R429C-expressing myotubes. Mitochondria with onion-shaped cristae were frequently observed (i.e., the enlargement and whirling of mitochondrial membranes, indicated by black arrows in the enlarged image 3 in Fig. 5a). In addition, the length of mitochondria with normal shapes (except for mitochondria with onion-shaped cristae) was significantly reduced by R429C (Fig. 5b and Table 2). Representative small mitochondria are indicated by black double arrowheads in the enlarged image 3 of Fig. 5a.

**Table 2:** Properties of mitochondria in R429C-expressing myotubes.

		Control	Wild-type STIM1	R429C
Mean length of mitochondria (µm)		1.72 ± 0.23 (81)	1.80 ± 0.22 (57)	1.20 ± 0.17 * (57)
[ATP] in 1 mg/ml myotube lysate (µM)		0.302 ± 0.006 (3)	0.305 ± 0.007 (3)	0.157 ± 0.008 * (3)
Expression level	Drp-1	1.000 ± 0.000 (3)	0.979 ± 0.061 (3)	1.196 ± 0.114 * (3)
	Mfn-1	1.000 ± 0.000 (3))	0.990 ± 0.048 (3)	0.872 ± 0.070 * (3)
	calcineurin	1.000 ± 0.000 (3)	0.992 ± 0.046 (3)	1.250 ± 0.043 * (3)
	CaMKII	1.000 ± 0.000 (3)	1.025 ± 0.027 (3)	1.263 ± 0.054 * (3)

The values are presented as the mean ± s.e.m. for the number of mitochondria or experiments shown in parentheses. For the expression level of proteins, the values were normalized to the mean values of the controls. *Significant difference compared with the control (*p* < 0.05).

To evaluate how the abnormalities in mitochondrial shape and length by R429C affect the function of mitochondria, the ATP level of R429C-expressing myotubes was measured using the lysate of R429C-expressing myotubes (Fig. 5c and Table 2). In agreement with the abnormalities in mitochondrial shape and length, the ATP level was dramatically reduced by R429C.

To examine the cause of the appearance of small mitochondria in R429C-expressing myotubes, the expression level of proteins that mediate mitochondrial fusion or fission was examined by immunoblot assays using the lysate of R429C-expressing myotubes. The expression of dynamin-1-like protein (Drp-1) that mediates mitochondrial fission was increased by R429C (Fig. 5d and Table 2). However, the expression of fusion-mediating mitofusin-1 (Mfn1) was decreased. In addition, the expression levels of calcineurin and Ca^2+^/calmodulin-dependent protein kinase II (CaMKII) that are known to activate Drp-1 by dephosphorylating or phosphorylating Drp-1, respectively, were examined. Expressions of both calcineurin and CaMKII were also increased by R429C (Fig. 5e and Table 2).

## Discussion

We found that R429C did not significantly affect the formation of skeletal myotubes during terminal differentiation (Fig. 1), suggesting that defects in myotube formations are not related to the human muscular hypotonia caused by R429C. This finding is in accordance with our previous reports showing no significant changes in myotube formations by knocking down either STIM1 or STIM2 (a major isoform of STIM1)^20,27,40^. Therefore these results suggest that STIM1 is not essential for the terminal differentiation of skeletal muscle. However, considering that STIM1 and STIM2 are functionally redundant in terms of terminal differentiation^41–44^, it is still possible that it plays a supportive role in this process.

In the present study, R429C bound to endogenous STIM1 (Fig. 2b), suggesting that R429C could oligomerize with endogenous STIM1. The oligomerization could affect two different events. First, the oligomerization could induce a slower activation of endogenous SOCE in R429C-expressing myotubes by interfering with the binding between endogenous STIM1 and Orai1 (as shown in Fig. 2a). Considering that effective oligomers of STIM1 to mediate SOCE are thought to be tetramers, hexamers, or other even-numbered oligomers^45^ and that the influence of R429C on endogenous SOCE was only found in the initiation phase of endogenous SOCE (but not in steady-state phase of endogenous SOCE), it is likely that the proportion of R429C to endogenous STIM1 in the oligomers is low. Second, the oligomerization could interfere with the binding between endogenous STIM1 and DHPR, which could remove the attenuation on DHPR-RyR1 coupling by endogenous STIM1 and result in the increase of intracellular Ca^2+^ movement in response to KCl in R429C-expressing myotubes (as shown in Fig. 3b). In addition, R429C showed homo-dimerization (Supplementary Fig. S4), suggesting that the oligomerization ability of STIM1 is not affected by the mutation at R429.

As previously reported^35^, R429C also does not mediate SOCE in skeletal myotubes. R429 is one of the residues of CC domain of STIM1 (Fig. 1a). It has been suggested that the destabilization of an α-helix in the CC3 domain by the mutation of R429 causes failure to bind to Orai1, which abolishes SOCE and subsequently causes insufficient immunity in human patients with R429C (i.e., a loss-of-function mutant)^35^. This could be a pathogenic factor in the human muscular hypotonia caused by R429C. However, considering that the Ca^2+^ entry mediated by SOCE plays a secondary role in skeletal muscle contraction, namely, not in the initiation but in the maintenance of the contraction^22–24^, a defect in SOCE by R429C is not fully responsible for the pathological mechanism of human muscular hypotonia associated with R429C.

Knock down of STIM1 in skeletal myotubes increases intracellular Ca^2+^ movement required for skeletal muscle contraction^20^, which is similar to the effect of R429C. However, unlike the knock down of STIM1, R429C induced abnormal intracellular Ca^2+^ distribution between the cytosol and the SR (higher Ca^2+^ levels in the cytosol and lower Ca^2+^ levels in the SR, Fig. 4) and increased the expression of Orai1 (Fig. 2c). Therefore, these differences suggest that in skeletal muscle, R429C is not only a loss-of-function but also a disease-causing mutant with a unique mechanism. The increase in cytosolic Ca^2+^ level could be a compensatory action for no mediation of SOCE by R429C, i.e., for the maintenance of the Ca^2+^ supply to the cytosol in response to no mediation of SOCE by R429C. The increased expression level of Orai1 by R429C (Fig. 2c) could be an attempt to compensate for no mediation of SOCE by R429C, although it was ineffective (Fig. 2a). The elimination of the attenuation of endogenous STIM1 on DHPR-RyR1 coupling by R429C (i.e., the increase of intracellular Ca^2+^ movement in response to KCl in Fig. 3b) could also contribute to the increase in the cytosolic Ca^2+^ levels and the reduction in Ca^2+^ amount in the SR. STIM2 attenuates SERCA1a activity during skeletal muscle contraction^40^, which is in a tug-of war relationship with STIM1 that activates SERCA1a activity and allows fine-regulations of SERCA1a activity in skeletal muscle^27^. In the present study, the expression level of STIM1 or STIM2 is not significantly changed by R429C (Fig. 2c) and R429 of STIM1 is not related to the binding region of STIM1 to SERCA1a^27,40^. Therefore it is likely that SERCA1a activity is not related to the changes in the higher level of cytosolic Ca^2+^ or the reduction of Ca^2+^ amount in the SR by R429C.

Despite of the dominant-negative effects of R429C over endogenous STIM1, the overall activity of RyR1 was not changed by R429C (Fig. 3c). It has been well-known that micromolar Ca^2+^ in the cytosol acts as an agonist of RyR1 by triggering Ca^2+^-induced Ca^2+^ release (CICR)^4,46^. Higher cytosolic Ca^2+^ level increases caffeine-sensitivity of RyR1^39,47^. In the present study, cytosolic Ca^2+^ level was increased by R429C (Fig. 4a), which allows an assumption that a higher activity of RyR1 in response to caffeine is possible. However, a reduction in the Ca^2+^ amount in the SR was also induced by R429C (Fig. 4b) and less amount of Ca^2+^ in the SR is usually favorable for a lower activity of RyR1^1,48^. It seems that a balance between these two opposite but possible events could result in no significant change in the overall activity of RyR1, as shown in Fig. 3c. This suggests that RyR1 activity in the presence of R429C could be dual-regulated by both Ca^2+^ in the SR and cytosol.

In the present study, dramatic changes in the mitochondria of skeletal myotubes were induced by R429C. First, R429C caused ultra-structural defects in mitochondria (Fig. 5a), namely, onion-shaped cristae (approximately 15~20% of the total mitochondria), also called ‘tubular parallel cristae’ or ‘concentric laminated bodies’^49^. Mitochondria with onion-shaped cristae have also been reported in the skeletal and cardiac muscle of human patients with cardiomyopathy and chronic congestive heart failure^50^. Second, R429C induced a significant decrease in ATP levels. ATP synthase in mitochondria forms long rows of dimers on tightly curved cristae ridges^51^. The onion-shaped cristae have no tightly curved ridges (Fig. 5a)^52^. Therefore, it is plausible that the lack of tightly curved ridges in the onion-shaped cristae could be the reason for the reduction in ATP levels. Third, the length of mitochondria with normal shapes was reduced by R429C. Additionally, the expression of Drp-1^53,54^, calcineurin^55^, or CaMKII^56^ that mediates mitochondrial fission or activates Drp-1 was increased by R429C. However, the expression of fusion-mediating Mfn1^57^ was decreased. Mitochondrial dysfunctions have been found in other skeletal myopathies, such as congenital muscular dystrophy^58^ and limb-girdle muscular dystrophy^59^, and in *mdx* mice^30,60^. However, there has been no report of a link between STIM1 and mitochondrial dysfunctions, with the notable exception of SOCE. Therefore this study results suggest a new clue that mitochondrial fusion and fission in skeletal muscle could be related to the STIM1-mediated intracellular Ca^2+^ movement and SOCE.

**Figure 6:**
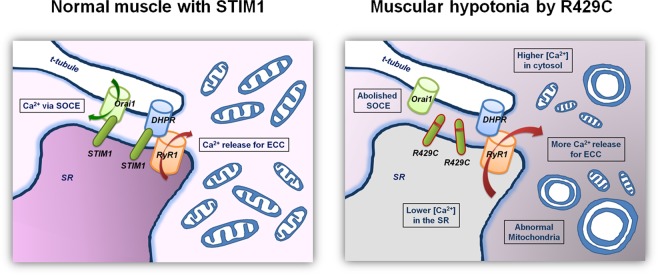
Possible pathologic mechanism of human skeletal muscular hypotonia by R429C. In skeletal muscle, R429C (a STIM1 mutant that causes human muscular hypotonia) does not mediate SOCE, induces more intracellular Ca^2+^ movement required for skeletal muscle contraction, lowers Ca^2+^ levels in the SR, and elevates cytosolic Ca^2+^ levels along with abnormal mitochondrial shapes and functions (less ATP), which could result in skeletal muscular hypotonia in humans who carry R429C. These findings suggest that, under physiological conditions, STIM1 in skeletal muscle regulates intracellular Ca^2+^ movement as well as extracellular Ca^2+^ entry (SOCE). ECC refers to EC coupling.

In conclusion, in skeletal muscle, STIM1 could regulate intracellular Ca^2+^ movement as well as SOCE. Serial Ca^2+^-related defects, such as no mediation of SOCE, increased intracellular Ca^2+^ movement for skeletal muscle contraction, and increased cytosolic Ca^2+^ levels along with mitochondrial abnormalities in shape and ATP level cause human skeletal muscular hypotonia. These phenomena could constitute a unique pathologic mechanism of skeletal muscular hypotonia in patients who possess R429C (depicted in Fig. 6).

## Materials and Methods

### Ethical approval

The methods were carried out in accordance with the guidelines and regulations of the College of Medicine at The Catholic University of Korea. All surgical interventions, including pre- and post-surgical animal care and the site where the animal work has taken place, were carried out in accordance with the Laboratory Animals Welfare Act, the Guide for Care and Use of Laboratory Animals, and the Guidelines and Policies for Rodent Survival Surgery approved by the Institutional Animal Care and Use Committee of the College of Medicine at The Catholic University of Korea. All experimental protocols were approved by the Committee of the College of Medicine at The Catholic University of Korea.

### cDNA construction

The mutation of R429 of STIM1 to C (R429C) was carried out using a site-directed mutagenesis kit (Agilent Technologies, Santa Clara, CA, U.S.A.), human STIM1 cDNA as a template (GenBank accession number: NM_003156.3), and a pair of complementary synthetic oligonucleotide primers containing the desired mutation (forward primer, 5′-CATTGCGGGAGCGCCTGCACTGCTGGCAACAGATC-3′; reverse primer, 5′-GATCTGTTGCCAGCAGTGCAGGCGCTCCCGCAATG-3′)^6,20,61^.

### Cell culture and the expression of R429C

Mouse primary skeletal myoblasts were derived from mouse skeletal muscle using a single-cell cloning method and were expanded and differentiated to myotubes, as previously described^20,61–63^. For the differentiation of skeletal myoblasts to myotubes, the myoblasts were re-plated on 96-well plates (for single-cell Ca^2+^ imaging of myotubes), on 6-well plates (for mitochondria observations), or on 10-cm plates (for other experiments) coated with Matrigel (BD Biosciences, Sparks Glencoe, MD, U.S.A.). After three days of culture in differentiation conditions, premature myotubes were transfected with an empty vector or cDNA encoding wild-type STIM1 or R429C (a mixture of 30 µl of FuGENE6 (Promega, Madison, WI, U.S.A.) and 20 μg of cDNA per 10-cm dish, or the same ratio of components in the well of other plates) for 3 hr. Mature myotubes were either imaged, observed, or disrupted at 36 hr post-transfection for further experiments, at which time approximately 60% of the myotubes had been transfected, as estimated by the CFP signal. All reagents for the cell cultures were obtained from Invitrogen (Thermo Fisher Scientific, Waltham, MA, U.S.A.).

### Co-immunoprecipitation and immunoblot assays

Mouse primary skeletal myotubes were solubilized in lysis buffer, as previously described^6,20,27,40,63,64^. For the co-immunoprecipitation assay^20,40,63^, solubilized myotube lysate (80 or 100 µg of total protein) was used. The immunoprecipitate was subjected to immunoblot assays with anti-DHPR, anti-STIM1, or anti-GFP antibody (Abcam, Cambridge, MA, U.S.A., for detecting CFP-R429C). For the immunoblot assays, the solubilized myotube lysate (10 μg of total protein) was subjected to SDS-PAGE (8, 10, or 12% gel)^6,20,27,40,63,64^. Anti-RyR1, anti-SERCA1a, anti-CSQ1, anti-CaM1, anti-MG29, anti-MG53, anti-JP1, and anti-JP2 antibodies were obtained from Affinity BioReagents (Golden, CO, U.S.A.). Anti-TRPC1, anti-TRPC3, anti-TRPC4, and anti-TRPC6 antibodies were obtained from Alomone Laboratories (Jerusalem, Israel). Anti-Orai1, anti-STIM1, anti-STIM2, and anti-α-actin antibodies were obtained from Abcam. Anti-Drp-1, anti-Mfn1, anti-calcineurin and anti-CaMKII antibodies were obtained from Santa Cruz Biotechnology (Paso Robles, CA, U.S.A.).

### qRT-PCR

Total RNA was obtained from myotubes. cDNAs were synthesized from the total RNA, as previously described^65^. qRT-PCR was performed using the following primers: MyoD (forward: 5′-GACCTGCGCTTTTTTGAGGACC-3′ and reverse: 5′-CAGGCCCACAGCAAGCAGCGAC-3′); myogenin (forward: 5′-TTGCTCAGCTCCCTCAACCAGGA-3′ and reverse: 5′- TGCAGATTGTGGGCGTCTGTAGG-3′); MHC (type II) (forward: 5′-GGCCAAAATCAAAGAGGTGA-3′ and reverse: 5′-CGTGCTTCTCCTTCTCAACC-3′); and GAPDH as a control (forward: 5′-AGGTCGGTGTGAACGGATTTG- 3′ and reverse: 5′-TGTAGACCATGTAGTTGAGGTCA-3′). A SYBR Green PCR kit with Taq DNA polymerase (Cosmo Genetech, Seoul, Korea) was used with a PCR thermocycler (Bio-Rad CFX96, Bio-Rad, Hercules, CA, U.S.A.). The PCR was conducted under the following conditions: denaturation at 94 °C for 10 sec, annealing at 58 °C for 10 sec, and extension at 62 °C for 15 sec. Samples of 20 ng cDNA were analyzed in triplicate. The obtained values were analyzed using a software (CFX manager Ver. 3.1, Bio-Rad), normalized to GAPDH, and presented as the fold-increase relative to the value of the control.

### Single-cell Ca^2+^ imaging of myotubes

Single-cell Ca^2+^ imaging of myotubes experiments were performed using an inverted-stage microscope (Nikon Eclipse TS100, Nikon Instruments, Inc., Melville, NY, U.S.A.) equipped with a 40x oil-immersion objective (NA 1.30), a high-speed monochromator with a 75 W xenon lamp (FSM150Xe, Bentham Instruments, Reading, Berkshire, U.K.), and a 12-bit charge-coupled device camera (DVC-340M-OO-CL, Digital Video Camera Company, Austin, TX, U.S.A.). Mouse primary skeletal myotubes were loaded with 5 μM fura-2-AM (Invitrogen) for the measurement of cytosolic [Ca^2+^] or fluo-4-AM (Invitrogen) for other measurements in an imaging buffer (25 mM HEPES, pH 7.4, 125 mM NaCl, 5 mM KCl, 2 mM KH_2_PO_4_, 2 mM CaCl_2_, 6 mM glucose, 1.2 mM MgSO_4_, and 0.05% BSA) at 37 °C for 45 min, as previously described^6,20,27,40,61,63,64^. Either KCl (60 mM) or caffeine (20 mM) was dissolved in the imaging buffer and applied to the myotubes via an auto-perfusion system (AutoMate Scientific, St. Berkeley, CA, U.S.A.). Before starting the single-cell Ca^2+^ imaging, images of myotubes were captured for size comparison (especially the width of myotubes). The data were displayed and analysed using image acquisition and analysis software (High-Speed InCyt Im1 and Im2, v5.29, Intracellular Imaging Inc., Cincinnati, OH, U.S.A.). To measure releasable Ca^2+^ from the SR or total cellular Ca^2+^ level, TG (2.5 μM) or ionomycin (5 μM) dissolved in dimethyl sulfoxide (DMSO, < 0.05%, no effect by itself) was applied to the myotubes, respectively, in the absence of extracellular Ca^2+^ to avoid the effect of extracellular Ca^2+^ entry. For the measurement of SOCE, Ca^2+^ in the SR was depleted with TG (2.5 μM) in the absence of extracellular Ca^2+^, and once the cytosolic Ca^2+^ level returned to baseline, 2 mM Ca^2+^ was added to the myotubes to measure SOCE. To analyse the Ca^2+^ movement obtained from single-cell Ca^2+^ imaging of myotubes, the peak amplitudes, which exhibited similar increases or decreases in peak areas, were measured. For long-term Ca^2+^ movements such as SOCE or the response to TG or ionomycin, the areas under the curves were analysed. To analyze the initial rate of SOCE, the slope at the rising phase of SOCE was examined by a linear equation that was obtained from a linear fitting of the rising phase of SOCE for initial 15 sec.^6,40,61,63^. Reagents for single-cell Ca^2+^ imaging of myotubes were obtained from Sigma-Aldrich (St. Louis, MO, U.S.A.).

### Width measurement

Measurements of the width in myotubes (one criterion that is used to evaluate the degree of skeletal myotube formation) were performed as previously described^6,20,27,61,64^. The width at the thickest part of each myotube was measured using the ImageJ program software (http://imagej.nih.gov/ij/).

### Immunocytochemistry and TEM

For immunocytochemistry assays, mouse primary skeletal myotubes were fixed in cold methanol (−20 °C) for 30 min and were permeabilized with 0.05% Tween 20 in phosphate-buffered saline (PBS) for 1 min, as previously described^6,20,27,63^. For TEM observations, the myotubes were fixed, embedded in epoxy resin (Epon 812), sectioned (70–80 nm) using an ultramicrotome (Ultracut UCT ultramicrotome, Leica, Buffalo Grove, IL, U.S.A.), and examined under TEM (JEM1010, JEOL Ltd., Peabody, MA, U.S.A.) at 60 kV, as previously described^6^. To analyse the length of mitochondria with normal, the length of mitochondria in a unit area (37.5 × 25.0 µm^2^) was measured using ImageJ software.

### ATP measurement

The ATP level of the mouse primary skeletal myotubes was measured using myotube lysate and an ATP bioluminescence assay kit (BioVision, Milpitas, CA, U.S.A.) according to the manufacturer’s protocol. Luminescence analysis was performed using a luminescence detector (SpectraMax L Microplate Reader and SoftMax Pro 5.4, Molecular Devices, San Jose, CA, U.S.A.).

### Statistical analysis

The results are presented as the mean ± s.e.m. calculated for the number of myotubes shown in parentheses in the tables or in the figure legends. The values were normalized to the mean value of the corresponding controls, except for those of cytosolic [Ca^2+^], mean length of mitochondria and [ATP]. Group differences were analysed using an unpaired *t*-test (GraphPad InStat, v2.04, GraphPad Software, San Diego, CA, U.S.A.). The differences were considered to be significant at *p* < 0.05. For qRT-PCR, the results are presented as the mean ± s.d. for triple experiments, and difference was considered to be considerable at more than 2-fold increase. The graphs were prepared using Origin v7 (OriginLab, Northampton, MA, U.S.A.).

## Supplementary information

Supplementary Information

## Data Availability

The data that support the findings of this study are available from the corresponding author upon reasonable request.
