# Supplementary material for: A muscular hypotonia-associated STIM1 mutant at R429 induces abnormalities in intracellular Ca2+ movement and extracellular Ca2+ entry in skeletal muscle
Source: Sci Rep. 2019 Dec 16;9:19140. doi: 10.1038/s41598-019-55745-z (PMC6915709; doi:10.1038/s41598-019-55745-z)

A muscular hypotonia-associated STIM1 mutant at R429 induces abnormalities in intracellular Ca^{2+} movement and extracellular Ca^{2+} entry in skeletal muscle

Jun Hee Choi, Mei Huang, Changdo Hyun, Mi Ri Oh, Keon Jin Lee, Chung-Hyun Cho, Eun Hui Lee

List of Supplementary Tables

Supplementary Table S1. mRNA level of MyoD, mygenin, and MHC in skeletal myotubes expressing wild-type STIM1 or R429C

Supplementary Table S2. Degree of co-immunoprecipitated R429C with endogenous STIM1 in R429C-expressing myotubes

Supplementary Table S3. Expression level of various proteins in skeletal myotubes expressing wild-type STIM1 or R429C

Supplementary Table S4. Degree of co-immunoprecipitated R429C with DHPR in R429C-expressing myotubes

List of Supplementary Figures

Supplementary Figure S1. Co-immunoprecipitation assay of CFP (tag) with endogenous STIM1

Supplementary Figure S2. Expression levels of various proteins in R429C-expressing myotubes

Supplementary Figure S3. Co-immunoprecipitation assay of DHPR with wild-type STIM1

Supplementary Figure S4. The full-length blots for proteins in the Fig. 2b of the manuscript

Supplementary Figure S5. The full-length blots for proteins in the Fig. 2c of the manuscript

Supplementary Figure S6. The full-length blots for proteins in the Fig. 3a of the manuscript

Supplementary Figure S7. The full-length blots for proteins in the Fig. 3d of the manuscript

Supplementary Figure S8. The full-length blots for proteins in the Fig. 5d of the manuscript

Supplementary Figure S9. The full-length blots for proteins in the Fig. 5e of the manuscript

Supplementary Table S1

mRNA level of MyoD, mygenin, and MHC in skeletal myotubes expressing wild-type STIM1 or R429C. Values in the histograms of Fig. 1c. The values were normalized to the mean values of the controls. The values are presented as the mean ± s.d. for triple experiments. Difference was considered to be considerable at more than 2-fold increase and there was no considerable difference.

	Control	Wild-type STIM1	R429C
**MyoD**	1.00 ± 0.02<br>(3)	1.22± 0.05<br>(3)	0.98 ± 0.14<br>(3)
**Myogenin**	1.00 ± 0.02<br>(3)	0.92 ± 0.05<br>(3)	1.07 ± 0.14<br>(3)
**MHC**	1.00 ± 0.15<br>(3)	1.00 ± 0.04<br>(3)	0.97 ± 0.25<br>(3)

Supplementary Table S2

Degree of co-immunoprecipitated R429C with endogenous STIM1 in R429C-expressing myotubes. Values in the histograms of Fig. 2b. The values are presented as the mean ± s.e.m. for the number of experiments shown in parentheses. *Significant difference compared with 'Without anti-STIM1 Ab' ($p < 0.05$).

	**Without anti-STIM1 Ab**	**With anti-STIM1 Ab**
**Degree of co-immunoprecipitated R429C with endogenous STIM1**	6.50 ± 3.43<br>(3)	69.58 ± 6.87 *<br>(3)

Supplementary Table S3

Expression level of various proteins in skeletal myotubes expressing wild-type STIM1 or R429C. Values in the histograms of Figs. 2c and 3d. The values were normalized to the mean values of the controls. The values are presented as the mean ± s.e.m. for the number of experiments shown in parentheses. *Significant difference compared with the control ($p < 0.05$).

	**Control**	**Wild-type STIM1**	**R429C**
**Orai1**	1.00 ± 0.00 (3)	1.04 ± 0.09 (3)	1.44 ± 0.06 * (3)
**STIM1 (endogenous)**	1.00 ± 0.00 (3)	0.99 ± 0.13 (3)	0.95 ± 0.15 (3)
**STIM2 (endogenous)**	1.00 ± 0.00 (3)	1.02 ± 0.16 (3)	0.97 ± 0.13 (3)
**JP1**	1.00 ± 0.00 (3)	0.97 ± 0.05 (3)	1.05 ± 0.06 (3)
**JP2**	1.00 ± 0.00 (3)	1.04 ± 0.08 (3)	1.06 ± 0.12 (3)
**MG29**	1.00 ± 0.00 (3)	0.96 ± 0.10 (3)	1.01 ± 0.06 (3)
**TRIM32**	1.00 ± 0.00 (3)	0.98 ± 0.09 (3)	0.98 ± 0.12 (3)
**RyR1**	1.00 ± 0.00 (3)	0.99 ± 0.11 (3)	1.02 ± 0.04 (3)
**DHPR**	1.00 ± 0.00 (3)	0.99 ± 0.09 (3)	1.03 ± 0.08 (3)
**SERCA1a**	1.00 ± 0.00 (3)	1.02 ± 0.07 (3)	0.99 ± 0.09 (3)

Supplementary Table S4

Degree of co-immunoprecipitated R429C with DHPR in R429C-expressing myotubes. Values in the histograms of Fig. 3a. The values are presented as the mean ± s.e.m. for the number of experiments shown in parentheses. Significant difference compared with 'Without anti-DHPR Ab' ($p < 0.05$) and there was no significant difference.

	**Without anti-DHPR Ab**	**With anti-DHPR Ab**
**Degree of co-immunoprecipitated R429C with DHPR**	5.42 ± 2.80<br>(3)	6.11 ± 5.88<br>(3)

Supplementary Figure S1

Co-immunoprecipitation assay of CFP (tag) with endogenous STIM1. (a) Lysate from the myotubes (that were transfected with empty vector and were used as a control) was immunoprecipitated with an anti-GFP antibody (to immunoprecipitate CFP) followed by an immunoblot assay of the immunoprecipitates with an anti-GFP or anti-STIM1 antibody. Endogenous STIM1 was not co-immunoprecipitated with CFP. A representative result is presented. The blots that were cropped from different gels were grouped and the full-length blots are presented. **(b)** Degree of co-immunoprecipitated endogenous STIM1 to the corresponding total protein is presented in the histograms. Significant difference compared with 'Without anti-GFP Ab' ($p < 0.05$) and there was no significant difference. **(c)** The values in **(b)** are presented as the mean ± s.e.m. for the number of experiments shown in parentheses.

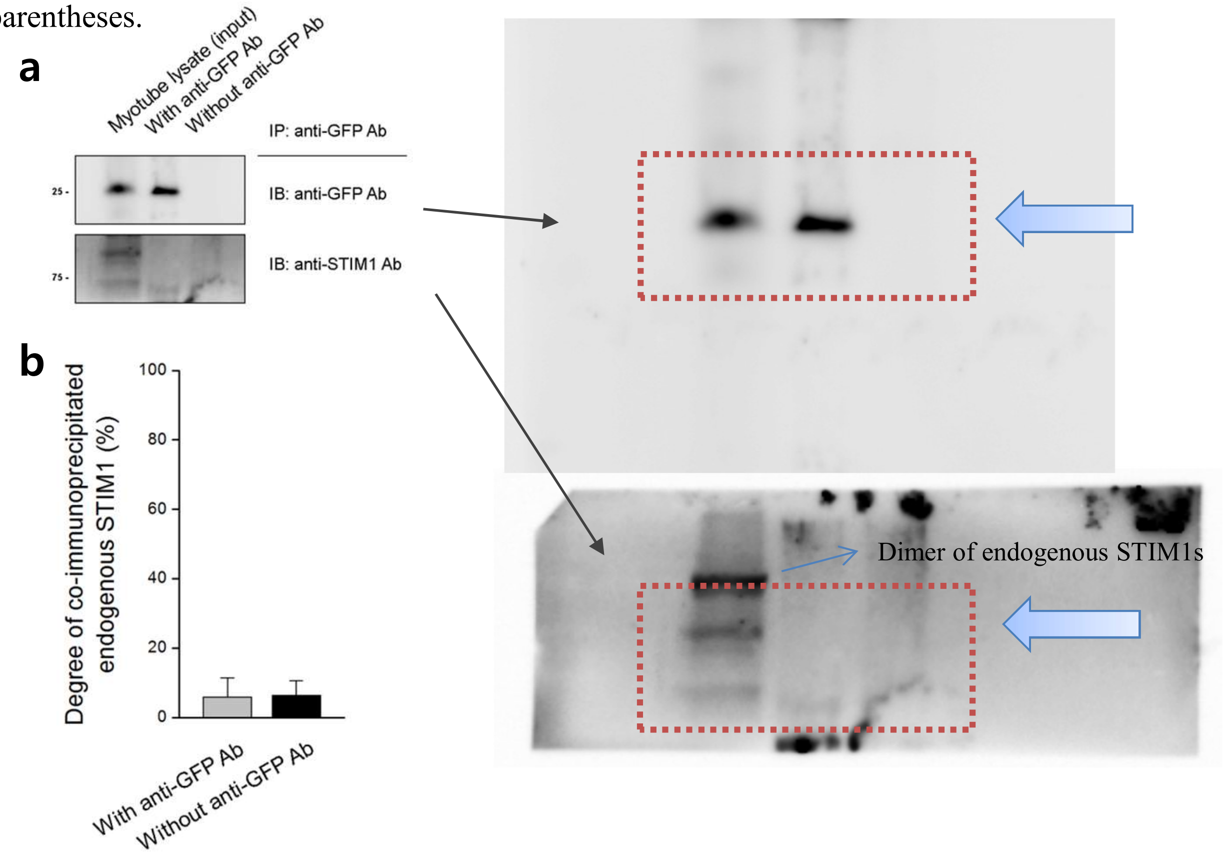

c

	With anti-GFP Ab	Without anti-GFP Ab
**Degree of co-immunoprecipitated endogenous STIM1 with CFP tag**	5.83 ± 5.64<br>(3)	6.41 ± 4.20<br>(3)

Supplementary Figure S2

Expression levels of various proteins in R429C-expressing myotubes. The lysate of R429C-expressing myotubes was subjected to immunoblot assays with antibodies against the seven indicated proteins that mediate extracellular Ca^{2+} entry or regulate Ca^{2+}-related skeletal muscle functions. α-actin was used as a loading control. Three independent experiments were conducted per protein. CSQ: calsequestrin; CaM1, calmodulin1; TRIM32, tripartite motif-containing protein 32. There was no significant change in their expression levels by R429C. The blots that were cropped from different gels were grouped and the full-length blots are presented.

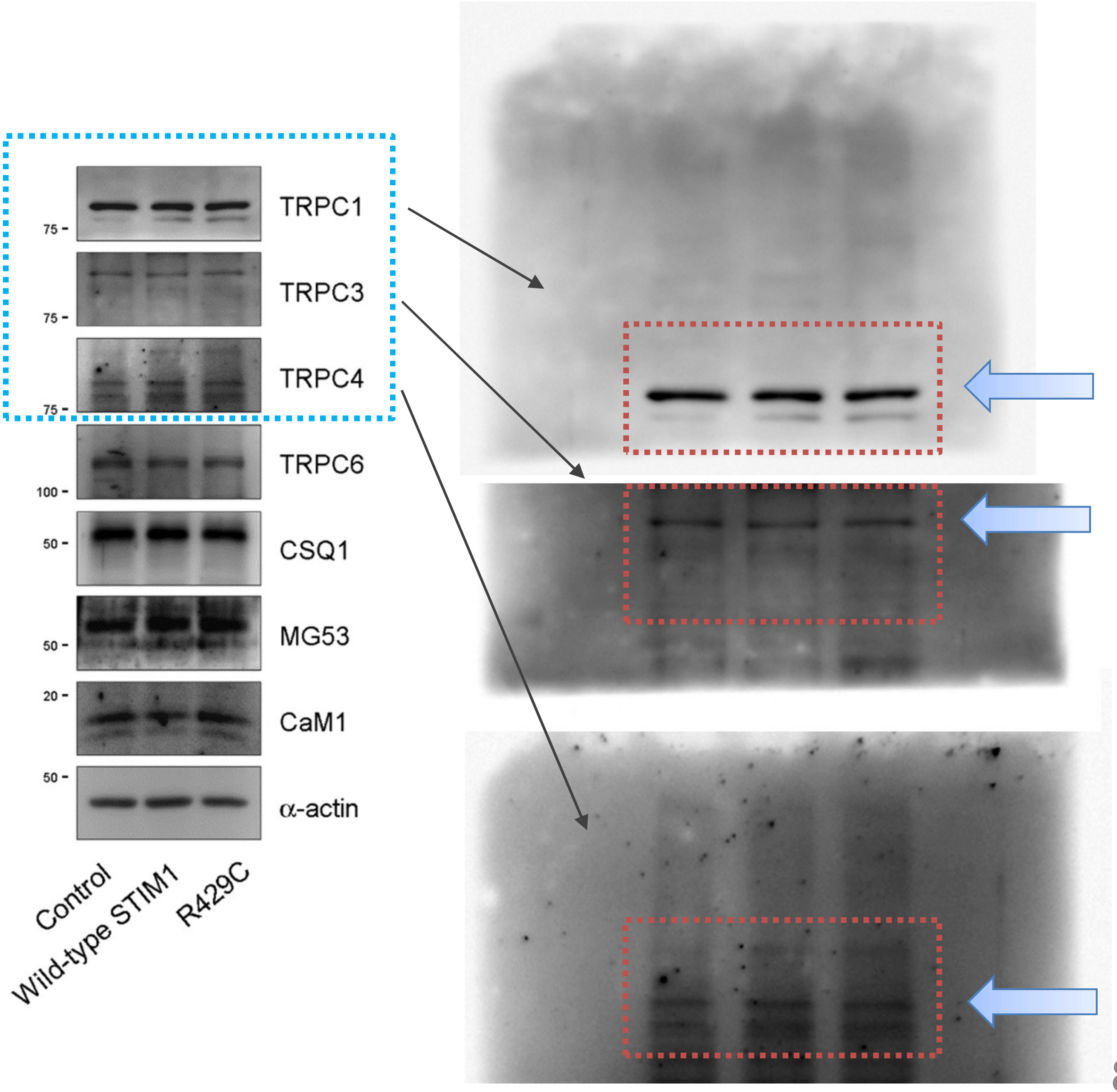

Supplementary Figure S2 (continued)

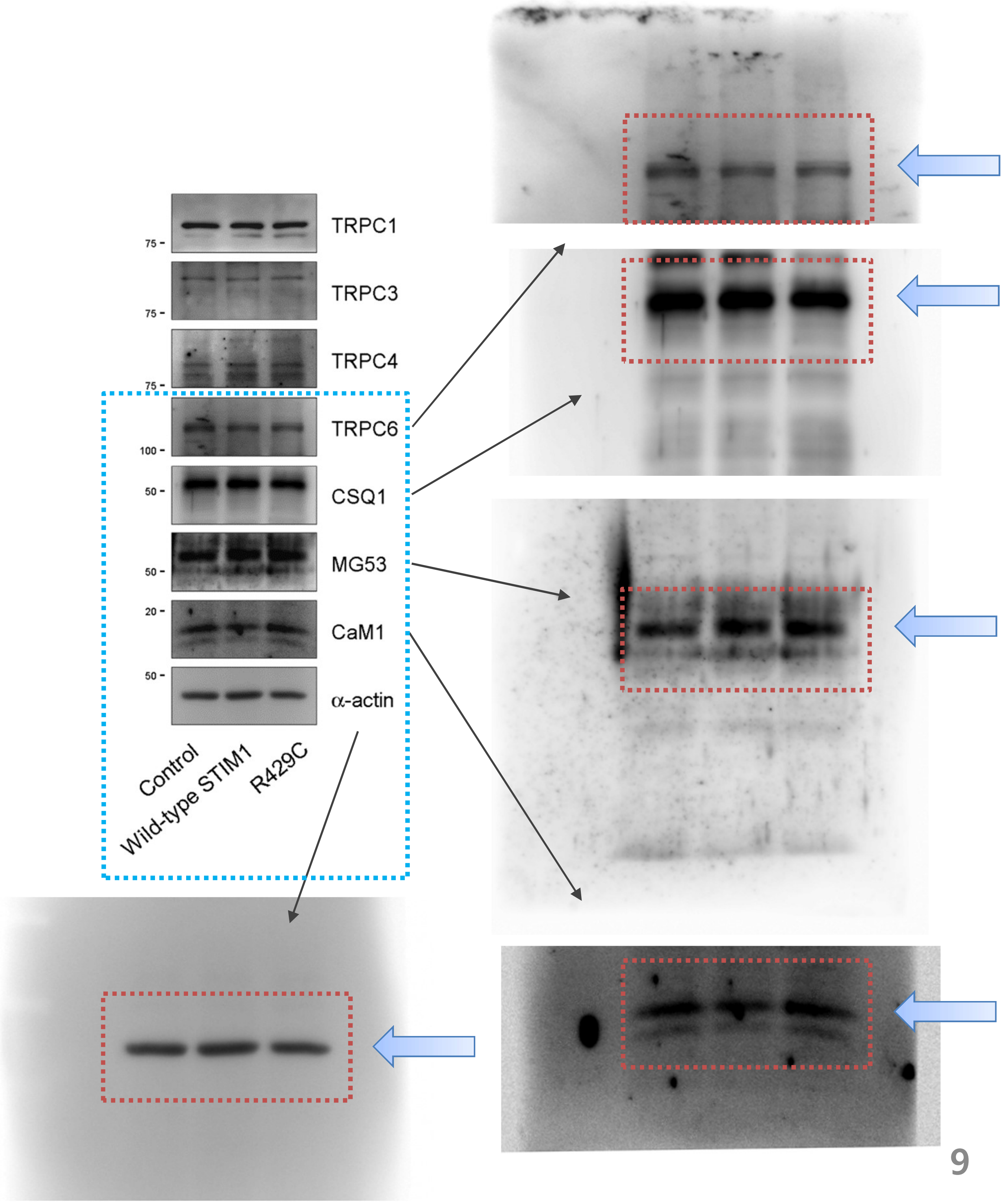

Supplementary Figure S3

Co-immunoprecipitation assay of DHPR with wild-type STIM1. Lysate from the myotubes was immunoprecipitated with an anti-STIM1 antibody followed by an immunoblot assay of the immunoprecipitates with an anti-STIM1, anti-DHPR or anti-RyR1 antibody. DHPR was co-immunoprecipitated with STIM1 (but not with RyR1). This data was adopted from a previously published article by our research group (Lee, K. J. *et al.* STIM1 negatively regulates Ca^{2+} release from the sarcoplasmic reticulum in skeletal myotubes. *Biochem J* **453**, 187-200, 2013).

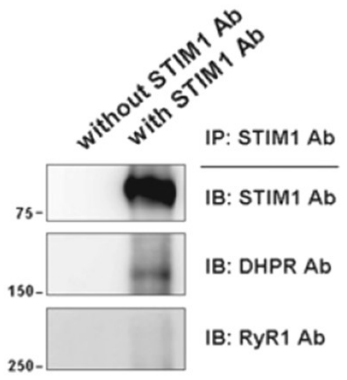

Supplementary Figure S4

The full-length blots for proteins in the Fig. 2b of the manuscript.

[Fig. 2b in the manuscript]

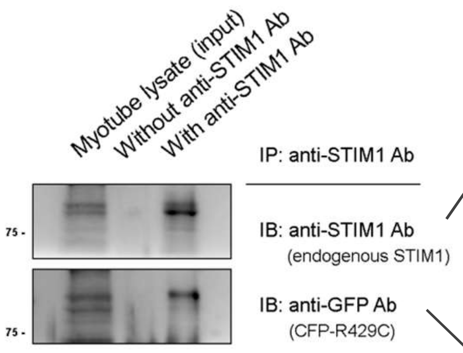

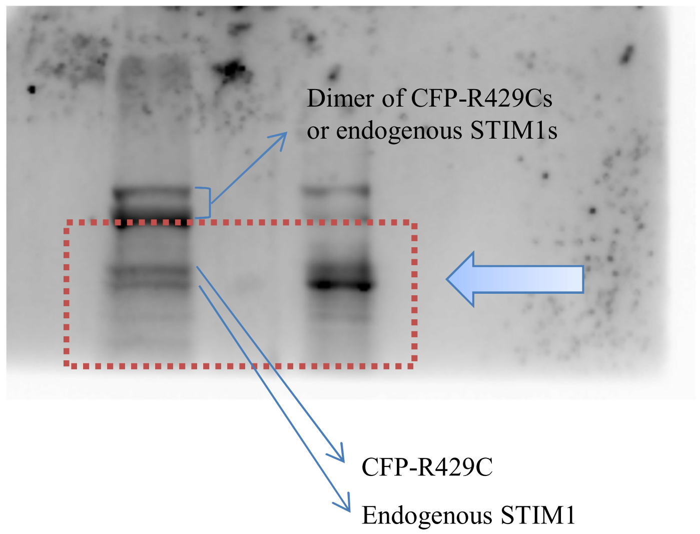

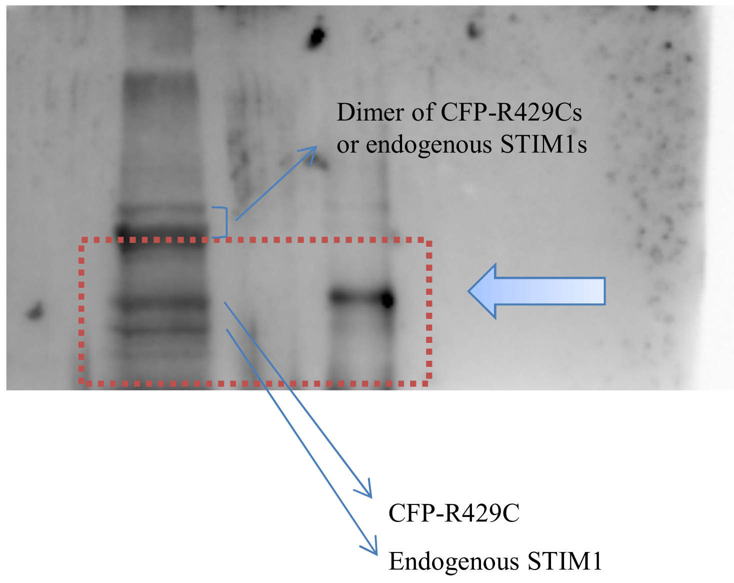

Supplementary Figure S5

The full-length blots for proteins in the Fig. 2c of the manuscript.

[Fig. 2c in the manuscript]

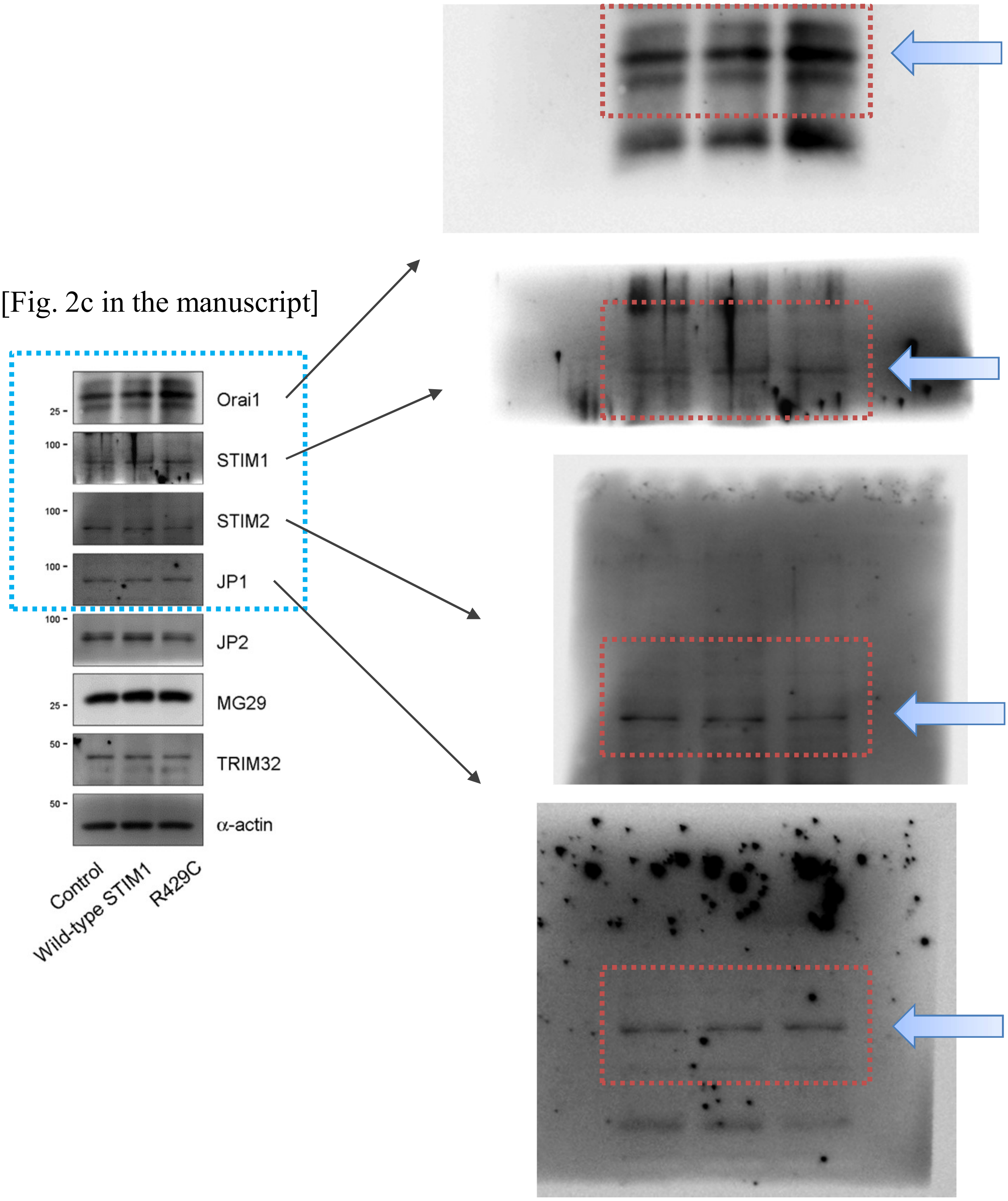

Supplementary Figure S5 (continued)

The full-length blots for proteins in the Fig. 2c of the manuscript.

[Fig. 2c in the manuscript]

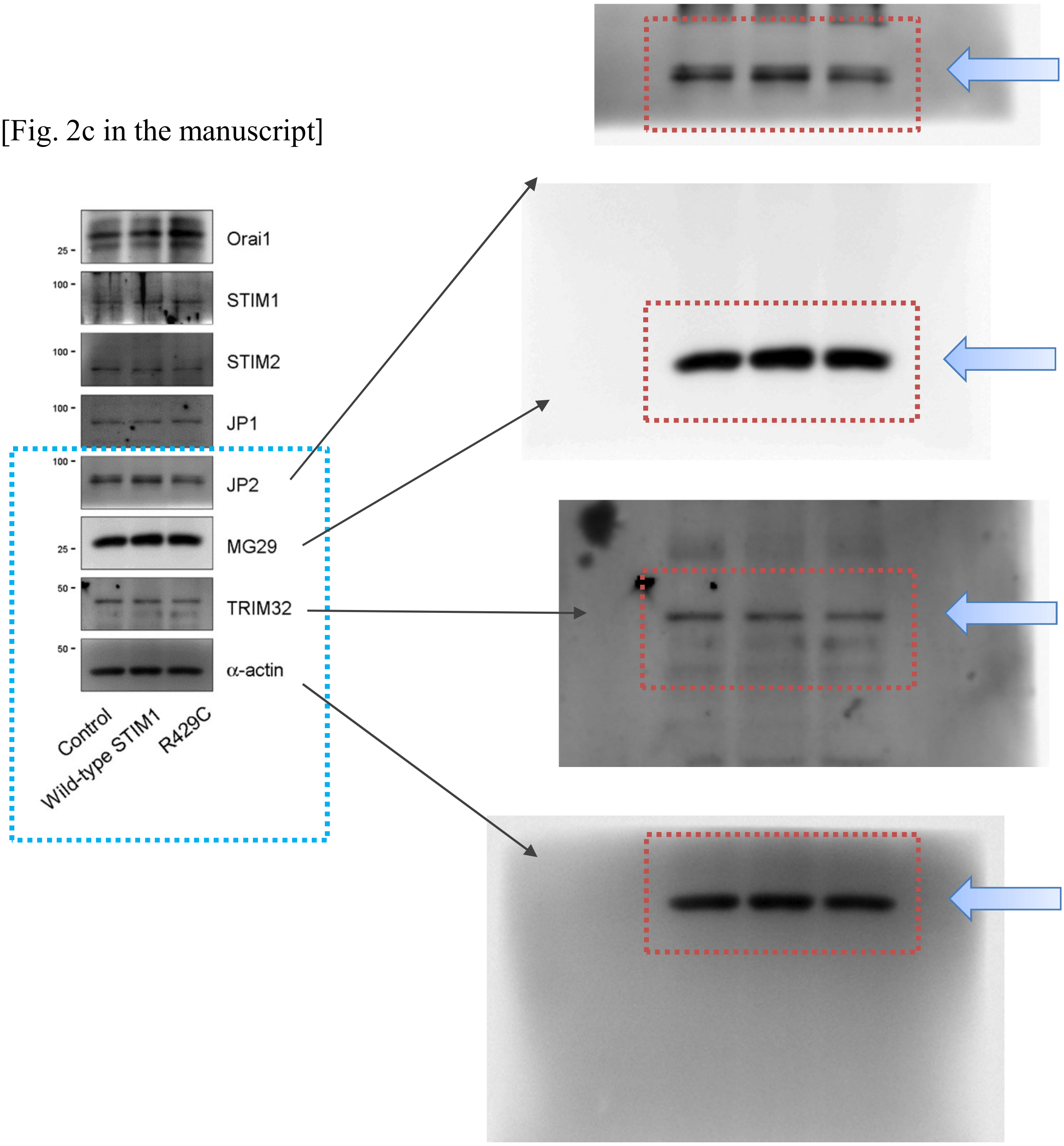

Supplementary Figure S6

The full-length blots for proteins in the Fig. 3a of the manuscript.

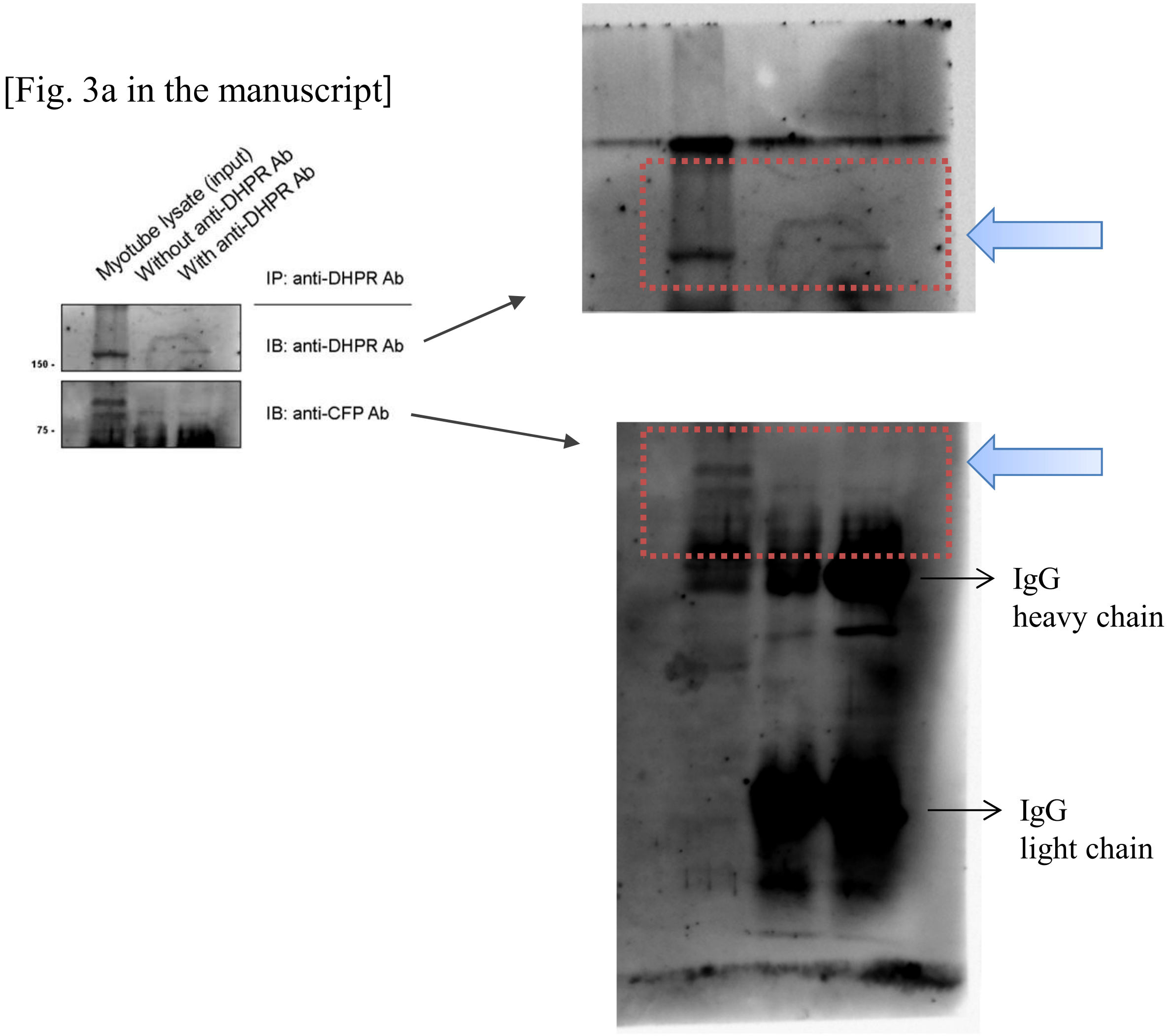

Supplementary Figure S7

The full-length blots for proteins in the Fig. 3d of the manuscript.

[Fig. 3d in the manuscript]

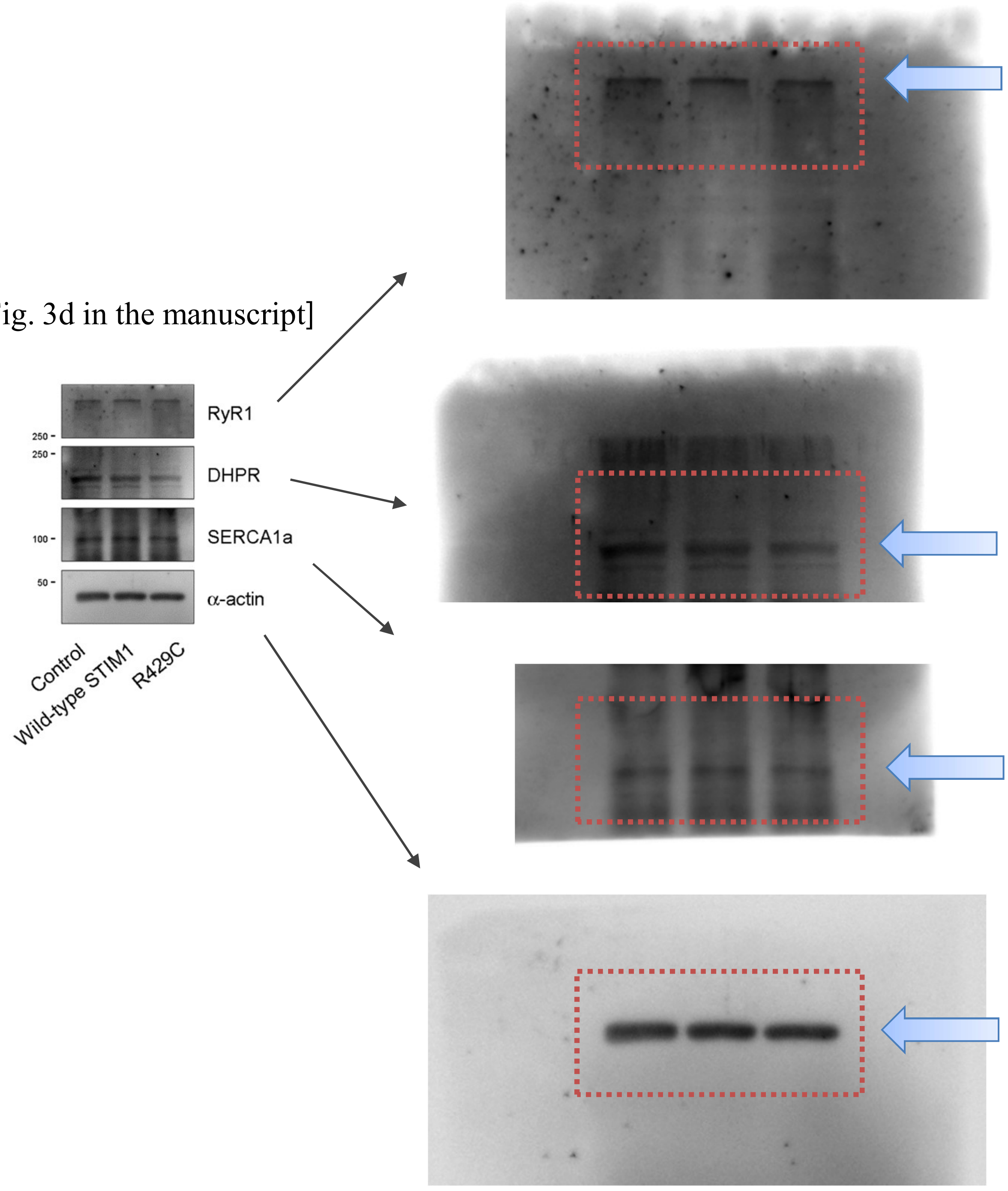

Supplementary Figure S8

The full-length blots for proteins in the Fig. 5d of the manuscript.

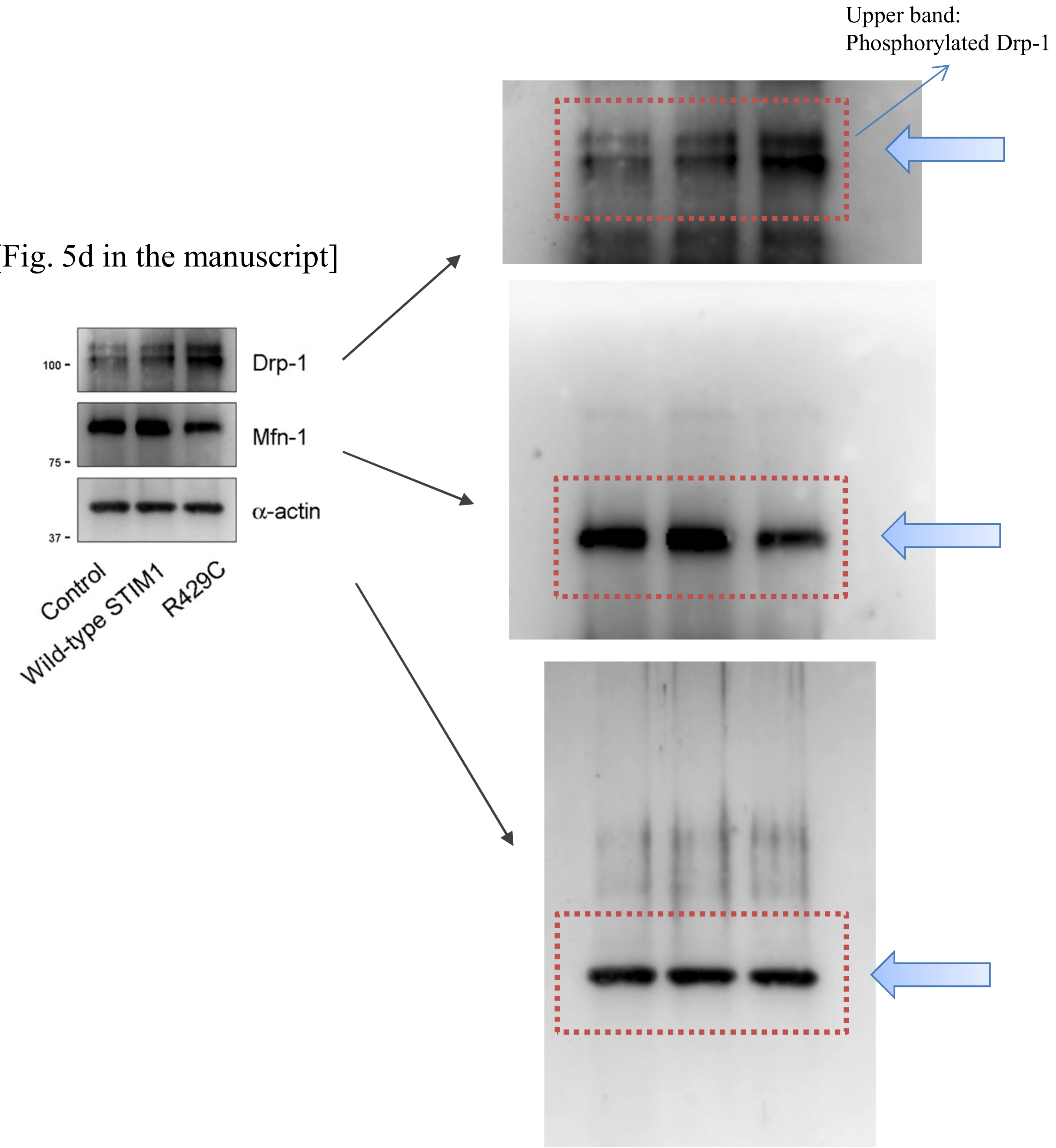

Supplementary Figure S9

The full-length blots for proteins in the Fig. 5e of the manuscript.

[Fig. 5e in the manuscript]

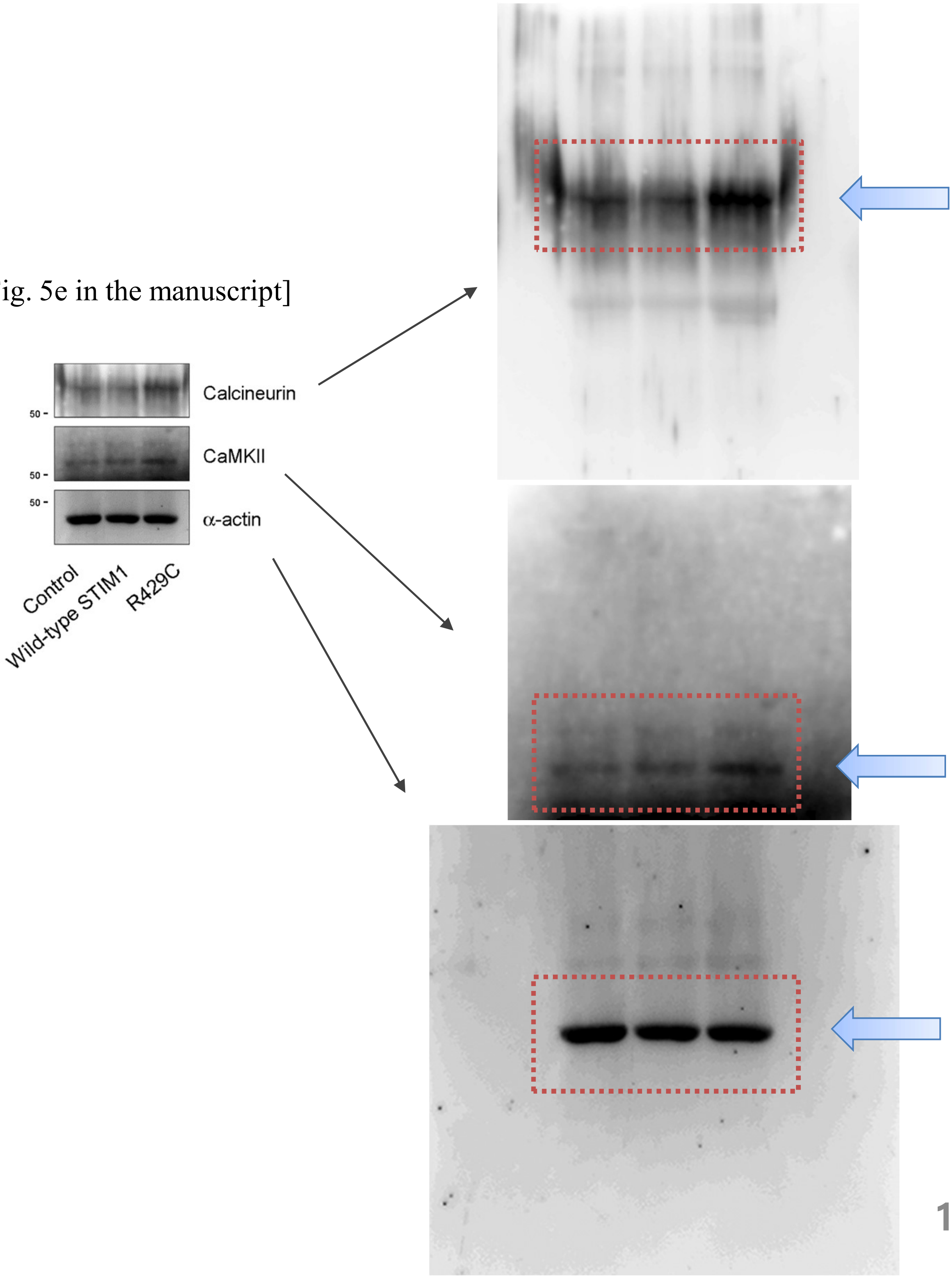

Supplement: Supplementary file 1 — Supplementary Information [file 41598_2019_55745_MOESM1_ESM.pdf]
